# Short-term outcomes and long-term quality of life of reconstruction methods after proximal gastrectomy: a systematic review and meta-analysis

**DOI:** 10.1186/s12885-024-11827-4

**Published:** 2024-01-10

**Authors:** Bailong Li, Yinkui Wang, Baocong Li, Fei Shan, Ziyu Li

**Affiliations:** Key laboratory of Carcinogenesis and Translational Research (Ministry of Education/Beijing), Gastrointestinal Cancer Center, Peking University Cancer Hospital and Institute, 52 Fucheng Road, Haidian District, Beijing, 100142 China

**Keywords:** Gastric cancer, Proximal gastrectomy, Digestive tract reconstruction, Short-term outcome, Quality of life, Meta-analysis

## Abstract

**Background:**

The optimal reconstruction method after proximal gastrectomy remains unclear. This systematic review and meta-analysis aimed to compare the short-term outcomes and long-term quality of life of various reconstruction methods.

**Methods:**

PubMed, Embase, Web of Science and Cochrane Library were searched to identify comparative studies concerning the reconstruction methods after proximal gastrectomy. The reconstruction methods were classified into six groups: double tract reconstruction (DTR), esophagogastrostomy (EG), gastric tube reconstruction (GT), jejunal interposition (JI), jejunal pouch interposition (JPI) and double flap technique (DFT). Esophagogastric anastomosis group (EG group) included EG, GT and DFT, while esophagojejunal anastomosis group (EJ group) included DTR, JI and JPI.

**Results:**

A total of 27 studies with 2410 patients were included in this meta-analysis. The pooled results indicated that the incidences of reflux esophagitis of DTR, EG, GT, JI, JPI and DFT were 7.6%, 27.3%, 4.5%, 7.1%, 14.0%, and 9.1%, respectively. The EG group had more reflux esophagitis (OR = 3.68, 95%CI 2.44–5.57, *P* < 0.00001) and anastomotic stricture (OR = 1.58, 95%CI 1.02–2.45, *P* = 0.04) than the EJ group. But the EG group showed shorter operation time (MD=-56.34, 95%CI -76.75- -35.94, *P* < 0.00001), lesser intraoperative blood loss (MD=-126.52, 95%CI -187.91- -65.12, *P* < 0.0001) and shorter postoperative hospital stay (MD=-2.07, 95%CI -3.66- -0.48, *P* = 0.01). Meanwhile, the EG group had fewer postoperative complications (OR = 0.68, 95%CI 0.51–0.90, *P* = 0.006) and lesser weight loss (MD=-1.25, 95%CI -2.11- -0.39, *P* = 0.004). For specific reconstruction methods, there were lesser reflux esophagitis (OR = 0.10, 95%CI 0.06–0.18, *P* < 0.00001) and anastomotic stricture (OR = 0.14, 95%CI 0.06–0.33, *P* < 0.00001) in DTR than the esophagogastrostomy. DTR and esophagogastrostomy showed no significant difference in anastomotic leakage (OR = 1.01, 95%CI 0.34–3.01, *P* = 0.98).

**Conclusion:**

Esophagojejunal anastomosis after proximal gastrectomy can reduce the incidences of reflux esophagitis and anastomotic stricture, while esophagogastric anastomosis has advantages in technical simplicity and long-term weight status. Double tract reconstruction is a safe technique with excellent anti-reflux effectiveness and favorable quality of life.

**Registration:**

This meta-analysis was registered on the PROSPERO (CRD42022381357).

**Supplementary Information:**

The online version contains supplementary material available at 10.1186/s12885-024-11827-4.

## Introduction

Gastric cancer (GC) is one of the leading digestive system malignancies. According to the data of GLOBOCAN in 2020, GC ranked the fifth in morbidity and the fourth in mortality among all malignancies worldwide [1]. In recent years, there has been an increasing trend in the incidence of early gastric cancer (EGC) and proximal gastric cancer, and proximal gastrectomy (PG), which is one of the function-preserving surgery has gained extensive attention [2, 3]. In the Japanese Gastric Cancer Treatment Guidelines 2021 (6th edition), PG is suggested for proximal gastric cancer with clinical stage of cT1N0 to preserve more than half of the distal stomach [4].

PG is capable of maintaining the volume of the remnant stomach and preserving the physiologic functions, such as the secretion of the intrinsic factors. These advantages can decrease the incidence of postoperative body weight loss and anemia compared to total gastrectomy (TG) [5]. However, due to the damage to the lower esophageal sphincter and the angle of His, the postoperative complications caused by PG, especially the reflux esophagitis (RE), may severely impair postoperative quality of life (QoL). Various functional digestive tract reconstruction methods after PG have been reported to reduce postoperative complications, such as double tract reconstruction, jejunum interposition and gastric tube reconstruction. But there is no consensus on the optimal reconstruction method after PG.

This systematic review and meta-analysis aim to comprehensively search comparative studies concerning reconstruction methods after PG and compare the postoperative short-term outcomes and long-term QoL.

## Materials and methods

This systematic review and meta-analysis was conducted following Preferred Reporting Items for Systematic Reviews and Meta-analyses (PRISMA) statements [6]. The protocol was registered on the PROSPERO website with the registration number CRD42022381357.

### Literature search strategy

Two authors (Bailong Li and Yinkui Wang) independently searched the databases of PubMed, Embase, Web of Science, and Cochrane Library to identify relevant studies published before December 2022. The search terms “proximal gastric cancer”, “proximal gastrectomy”, “reconstruction”, “anastomosis” and Medical Subject Headings (MeSH) “surgical anastomosis” were used to form the search strategies. Moreover, the references cited in the included articles and previously published reviews were manually searched to identify additional relevant studies. If duplicated studies were published by the same authors with an accumulated number of patients or updated follow-up, only the latest article was included.

### Inclusion and exclusion criteria

The inclusion criteria were: (1) Patients were pathologically diagnosed with GC or adenocarcinoma of the esophagogastric junction (AEG); (2) Patients underwent laparoscopic proximal gastrectomy, laparoscopic-assisted, or open proximal gastrectomy; (3) The reconstructive techniques included using linear stapler, circular stapler, and hand-sewn anastomosis. (4) Comparative studies concerning two or more digestive tract reconstruction methods after PG.

The exclusion criteria were: (1) Patients were pathologically diagnosed with other digestive tract tumors, such as gastrointestinal stromal tumors; (2) Single-arm study; (3) Case reports or all the cases added up less than 10; (4) Review articles, meeting abstracts and comments; (5) Studies that did not provide necessary data for statistical analysis.

### Outcome definition

The postoperative short-term outcomes included postoperative complications, such as anastomotic leakage and anastomotic stricture. The surgical and postoperative recovery indicators were also collected, including intraoperative blood loss, operation time and postoperative hospital stay. The long-term QoL included postoperative symptoms such as reflux, dysphagia and distention and nutritional status. The nutritional status included body weight loss and hematological indexes, such as hemoglobin, albumin, and vitamin B12. The reflux esophagitis was confirmed by endoscopy 12 months after surgery and classified by Los Angeles classification [7]. Los Angeles classification degree B or more severe degrees were recorded and analyzed. The degree of food residue was evaluated according to the RGB classification, and a grade ≥ 2 was recorded [8].

### Data extraction and quality assessment

Two authors (Bailong Li and Yinkui Wang) independently screened titles, abstracts, and full texts of studies. Disagreement between the two reviewers was resolved through a discussion with another author (Fei Shan).

Data of studies were extracted onto Excel spreadsheets by two authors (Bailong Li and Baocong Li). The following data were extracted: (1) Study characteristics, including the first author, country, study period, year of publication, study design, and the number of patients in each group; (2) Patient characteristics, including age, gender, tumor location, surgical approach, the reconstruction methods, the reconstruction techniques and the size of the remnant stomach; (3) Short-term outcomes, including the number of the overall postoperative complications, the number of specific complications; the intraoperative indexes, such as intraoperative blood loss, operation time, and postoperative hospital stay; (4) Long-term QoL, including the number of patients with postoperative long-term symptoms, the body weight loss, and hematological indexes 12 months after surgery.

Two authors (Bailong Li and Baocong Li) independently assessed the quality of included studies. The disagreement between the two authors was resolved by discussion. Newcastle-Ottawa quality assessment scale (NOS) was used to evaluate the risk of bias in nonrandomized studies [9]. Considering 8 items associated with selection, comparability and exposure, and the total score was calculated by summing the values. The total score was 9. Cochrane Handbook for Systematic Reviews of Interventions was used to assess the risk of bias in randomized controlled trials (RCTs) [10]. The risk of bias consists of selection bias, performance bias, detection bias, attrition bias, reporting bias, and other bias. Each term was judged as “low risk”, “high risk” or “unclear risk”.

### Statistical analysis

Review Manager (RevMan) (Version 5.4, The Cochrane Collaboration, 2020) was used to perform the statistical analysis. Continuous variables were pooled using the mean difference (MD) with a 95% confidence interval (95% CI), and discontinuous variables were pooled with an odds ratio (OR) with 95% CI. Heterogeneity was tested with Cochran’ Q test and quantified with the I^2^ inconsistency test. Heterogeneity was graded as low (I^2^ < 25%), moderate (I^2^ = 25–50%), and high (I^2^ > 50%). If I^2^>50%, a random effects model was adopted, otherwise, a fixed effects model was used. Sensitivity analysis was performed to identify the sources of heterogeneity by removing one study in meta-analysis at a time. Subgroup analysis was conducted to adjust for the heterogeneity if appropriate. Publication bias was explored graphically with funnel plots to detect asymmetry and outliers. In some studies, mean value and standard deviation were estimated from the median, range, and sample size according to Hozo’s report [11]. A *p*-value<0.05 is considered statistically significant.

## Results

### Search results and study characteristics

**Fig. 1:**
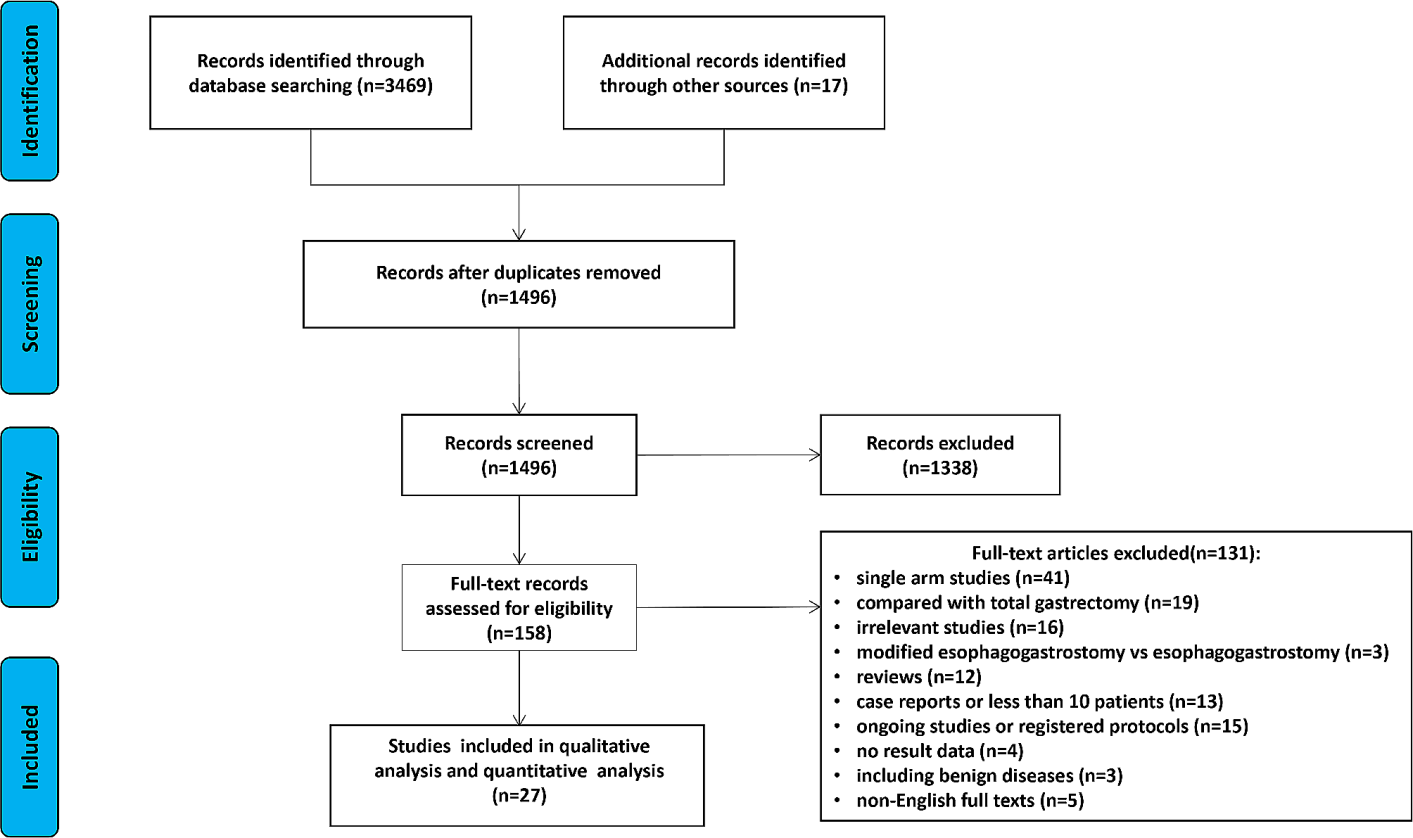
PRISMA flow chart

There were 3469 studies initially identified through searching databases. In addition, seventeen studies were manually added according to the reference lists of published reviews and articles. A total of 1496 studies remained after removing duplicates. Among them, a total of 1338 studies were excluded through screening titles and abstracts, and the remaining 158 studies were further assessment by full-text review. Finally, twenty-seven studies were included for qualitative analysis and quantitative analysis (meta-analysis) [12–38]. The PRISMA flow chart for the literature selection process is presented in Fig. 1.

A total of 2410 patients were included in the 27 studies. These studies were published from 1999 to 2021, including 3 RCTs and 24 retrospective studies. All the studies were reported by Asian authors. Among the 27 studies, sixteen were from Japan, ten were from China and 1 from Korea. The characteristics of included studies are shown in Table 1.

**Table 1:** Characteristics of included studies

Authors	Country	Study period	Publication year	Design	Tumor location	Approach	Groups	Reconstruction techniques	Size of remnant stomach	Number of patients
Aburatani T, et al.	Japan	2005–2014	2017	RS	U/EGJ	LAPG	DTR/EG	Circular	-	19/22
Adachi Y, et al.	Japan	1992–1998	1999	RS	U	OPG	GT/JI	Circular	One-third	14/16
Chen X, et al.	China	2009–2010	2012	RS	EGJ	OPG	GT/EG	-	30–60%	35/41
Eom B, et al.	Korea	2013–2017	2021	RS	U	LPG	DTR/EG	Linear/ Hand- sewn	-	58/45
Hu L, et al.	China	2006–2019	2021	RS	U	OPG + LPG	JI/EG	-	-	43/39
Ichikawa D, et al.	Japan	1992–1999	2001	RS	U	-	JI/EG	-	-	13/13
Isobe T, et al.	Japan	1989–2008	2014	RS	U	-	EG/JI/JPI	-	-	66/23/12
Ji X, et al.	China	2014–2019	2021	RS	U/EGJ	OPG	DTR/EG	Circular	More than half	25/39
Li L, et al.	China	2000–2009	2011	RS	-	-	EG/JI/GT	Circular	-	50/26/44
Li Z, et al.	China	2015–2017	2019	RCT	EGJ	OPG	DTR/JI	-	-	98/103
Masuzawa T, et al.	Japan	1998–2005	2014	RS	U	-	JI/EG	-	-	32/49
Miyauchi W, et al.	Japan	2010–2018	2020	RS	U	LPG	DTR/EG	Linear/Circular	-	24/23
Nakamura M, et al.	Japan	1999–2011	2014	RS	U	-	EG/JI/JPI	Circular	Half	55/25/12
Nomura E, et al.	Japan	2012–2016	2019	RS	-	LPG	DTR/JI	Circular	Half	15/15
Shiraishi N, et al.	Japan	1993–1999	2002	RS	U	OPG	GT/JI	Circular	-	14/17
Takagawa R, et al.	Japan	2000–2008	2010	RCT	U	-	JI/JPI	Circular	-	19/19
Tokunaga M, et al.	Japan	1996–2005	2008	RS	-	-	JI/EG	Circular	-	40/36
Toyomasu Y, et al.	Japan	2000–2014	2016	RS	U	LAPG	GT/JI	Circular	One-third	84/40
Wang X, et al.	China	2018–2020	2021	RS	U	OPG + LPG + RPG	DTR/EG	-	-	89/83
Yasuda A, et al.	Japan	2001–2011	2015	RS	U	LAPG + OPG	GT/JI	Circular	-	25/21
Zeng C, et al.	China	2000–2012	2014	RS	U	-	JI/EG	-	-	28/40
Zhang B, et al.	China	2011–2011	2013	RS	U	OPG	JI/EG	-	-	30/30
Zhang Z, et al.	China	2010–2011	2013	RCT	U	-	JI/EG	Circular	-	41/41
Zhao Q, et al.	China	2004–2008	2015	RS	EGJ	OPG	JI/EG	Circular	-	266/252
Kumamoto, et al.	Japan	2011–2016	2021	RS	U	OPG	DFT/JI	Hand- sewn/Circular	-	14/20
Sakuramoto, et al.	Japan	2005–2008	2009	RS	U	LAPG	DTR/ EG	Linear	-	10/26
Seshimo, et al.	Japan	1999–2012	2013	RS	U	-	JI/EG	-	-	18/26

*RS* retrospective study *RCT* randomized controlled trail *U* upper *EGJ* esophagogastric junction *LAPG* laparoscopic-assisted proximal gastrectomy *OPG* open proximal gastrectomy *LPG* laparoscopic proximal gastrectomy *DTR* double tract reconstruction *EG* esophagogastrostomy *JI* jejunal interposition *GT* gastric tube reconstruction *JPI* jejunal pouch interposition

### Results of qualitative analysis

The reconstruction methods after PG were classified into six groups: double tract reconstruction (DTR), esophagogastrostomy (EG), gastric tube reconstruction (GT), jejunal interposition (JI), jejunal pouch interposition (JPI) and double flap technique (DFT). The details of postoperative outcomes are shown in Table 2.

**Table 2:** Qualitative analysis for various reconstruction methods

Author	Reflux esophagitis	Stricture	Anastomotic leakage	Early complications	Residual food
DTR					
Aburatani et al.	2/19 (10.5%)	0/19 (0%)	0/19 (0%)	1/19 (5.3%)	1/19 (5.3%)
Eom B et al.	1/58 (1.7%)	5/58 (8.6%)	-	-	-
Ji X et al.	0/25 (0%)	-	2/25 (8.0%)	6/25 (24.0%)	-
Li Z et al.	3/103 (2.9%)	-	1/103 (1.0%)	2/103 (1.9%)	-
Miyaochi et al.	3/24 (12.5%)	0/24 (0%)	4/24 (16.7%)	5/24 (20.8%)	-
Nomura E et al.	1/15 (6.7%)	2/15 (13.3%)	0/15 (0%)	3/15 (20.0%)	2/15 (13.3%)
Wang X et al.	14/89 (15.7%)	1/89 (1.1%)	1/89 (1.1%)	33/89 (37.1%)	-
Sakuramoto et al.	2/8 (25%)	1/8 (12.5%)	0/10 (0%)	2/10 (20.0%)	-
Total	26/341 (7.6%)	9//213 (4.2%)	8/285 (2.8%)	52/285 (18.2%)	3/34 (8.8%)
EG					
Aburatani et al.	5/22 (22.7%)	6/22 (27.3%)	0/22 (0%)	6/22 (27.3%)	13/22 (59.1%)
Chen X et al.	3/41 (7.3%)	9/41 (22.0%)	0/41 (0%)	23/41 (56.1%)	-
Eom B et al.	5/45 (11.1%)	11/45 (24.4%)	-	-	-
Hu L et al.	16/39 (41.0%)	2/39 (5.1%)	-	3/39 (7.7%)	-
Ji X et al.	3/39 (7.7%)	-	4/39 (10.3%)	10/39 (25.6%)	-
Masuzawa et al.	9/49 (18.4%)	2/49 (4.1%)	0/49 (0%)	4/49 (8.2%)	-
Miyaochi et al.	17/23 (73.9%)	7/23 (30.4%)	3/23 (13.0%)	17/23 (73.9%)	-
Tokunaga et al.	11/36 (30.6%)	-	0/36 (0%)	3/36 (8.3%)	-
Wang X et al.	58/83 (69.9%)	14/83 (16.9%)	1/83 (1.2%)	21/83 (25.3%)	-
Zhang Z et al.	10/41 (24.4%)	0/41 (0%)	0/41 (0%)	0/41 (0%)	-
Zhao Q et al.	-	-	3/252 (1.2%)	21/252 (8.3%)	-
Isobe T et al.	12/66 (18.2%)	2/66 (3.0%)	1/66 (1.5%)	8/66 (12.1%)	-
Li L et al.	-	2/50 (4.0%)	1/50 (2.0%)	-	-
Nakamura et al.	12/55 (21.8%)	12/64 (18.8%)	0/64 (0/%)	2/55 (3.6%)	12/55 (21.8%)
Zeng C et al.	7/40 (17.5%)	-	-	1/40 (2.5%)	-
Zhang B et al.	-	-	2/30 (6.7%)	7/30 (23.3%)	-
Kim MC et al.	4/26 (15.4%)	4/26 (15.4%)	1/26 (3.8%)	6/26 (23.1%)	10/26 (38.5%)
Han W et al.	6/30 (20.0%)	4/30 (13.3%)	1/30 (3.3%)	7/30 (23.3%)	20/30 (66.7%)
Seshimo et al.	10/46 (21.7%)	5/46 (10.9%)	-	4/46 (8.7%)	-
Ichikawa et al.	3/13 (23.1%)	0/13 (0%)	0/13 (0%)	0/13 (0%)	-
Sakuramoto et al.	4/20 (20.0%)	0/20 (0/%)	2/26 (8.7%)	2/26 (7.7%)	-
Total	195/714(27.3%)	80/638 (12.5%)	19/865 (2.2%)	145/957 (15.2%)	55/133 (41.4%)
GT					
Adachi Y et al.	1/14 (7.1%)	1/14 (7.1%)	0/14 (0%)	2/14 (14.3%)	-
Chen X et al.	0/35 (0%)	4/35 (11.4%)	0/35 (0%)	10/35 (28.6%)	-
Toyomasu et al.	3/84 (3.6%)	11/84 (13.1%)	1/84 (1.2%)	12/84 (14.3%)	-
Yasuda et al.	3/22 (13.6%)	5/23 (21.7%)	0/25 (0%)	4/25 (16.0%)	4/22 (18.2%)
Li L et al.	-	8/44 (18.2%)	4/44 (9.1%)	-	-
Total	7/155 (4.5%)	29/200 (14.5%)	5/202 (2.5%)	28/158 (17.7%)	4/22 (18.2%)
JI					
Adachi Y et al.	0/16 (0%)	1/16 (6.3%)	0/16 (0%)	1/16 (6.3%)	-
Hu L et al.	7/43 (16.3%)	3/43 (7.0%)	-	9/43 (20.9%)	-
Kumamoto et al.	0/17 (0%)	0/20 (0%)	0/20 (0%)	0/20 (0%)	7/17 (41.2%)
Li Z et al.	3/98 (3.1%)	-	-	2/98 (2.0%)	-
Masuzawa et al.	5/32 (15.6%)	1/32 (3.1%)	0/32 (0%)	3/32 (9.4%)	-
Nomura E et al.	1/15 (6.7%)	2/15 (13.3%)	0/15 (0%)	2/15 (13.3%)	4/15 (26.7%)
Takagawa et al.	3/19 (15.8%)	4/19 (21.1%)	3/19 (15.8%)	6/19 (31.6%)	-
Tokunaga et al.	2/40 (5.0%)	-	0/40 (0%)	6/40 (15.0%)	-
Toyomasu et al.	2/40 (5.0%)	5/40 (12.5%)	0/40 (0%)	5/40 (12.5%)	-
Yasuda et al.	0/17 (0%)	2/20 (10.0%)	2/21 (9.5%)	6/21 (28.6%)	10/17 (58.8%)
Zhang Z et al.	1/41 (2.4%)	0/41 (0%)	0/41 (0%)	0/41 (0%)	-
Zhao Q et al.	29/244 (11.9%)	-	3/266 (1.1%)	23/266 (8.6%)	-
Isobe T et al.	3/23 (13.0%)	0/23 (0%)	3/23 (13.0%)	6/23 (26.1%)	-
Li L et al.	-	1/26 (3.8%)	1/26 (3.9%)	-	-
Nakamura et al.	0/22 (0%)	7/25 (28.0%)	1/25 (4.0%)	5/25 (20.0%)	7/22 (31.8%)
Zeng C et al.	3/28 (10.7%)	-	-	2/28 (7.1%)	-
Zhang B et al.	-	-	2/30 (6.7%)	8/30 (26.7%)	-
Seshimo et al.	2/18 (11.1%)	1/18 (5.6%)	-	4/18 (22.2%)	-
Ichikawa et al.	2/13 (15.4%)	2/13 (15.4%)	-	1/13 (7.7%)	-
Total	34/482 (7.1%)	29/351 (8.3%)	15/614 (2.4%)	89/788 (11.3%)	28/71 (39.4%)
JPI					
Takagawa et al.	3/19 (15.8%)	2/19 (10.5%)	1/19 (5.3%)	1/19 (5.3%)	-
Isobe T et al.	2/12 (16.7%)	1/12 (8.3%)	1/12 (8.3%)	4/12 (33.3%)	-
Nakamura et al.	1/12 (8.3%)	1/12 (8.3%)	0/12 (0%)	3/12 (25.0%)	11/12 (91.7%)
Total	6/43 (14.0%)	4/43 (9.3%)	2/43 (4.7%)	8/43 (18.6%)	11/12 (91.7%)
DFT					
Kumamoto et al.	1/11 (9.1%)	0/14 (0%)	1/14 (7.1%)	2/14 (14.3%)	4/11 (36.3%)
Total	1/11 (9.1%)	0/14 (0%)	1/14 (7.1%)	2/14 (14.3%)	4/11 (36.3%)

### Double tract reconstruction

There were eight studies investigating DTR, and a total of 341 patients were included [12, 15, 19, 22, 24, 25, 27, 33]. The incidence of postoperative RE, stricture, anastomotic leakage, early complications and residual food were 7.6%, 4.2%, 2.8%, 18.2% and 8.8%, respectively.

### Esophagogastrostomy

Twenty studies reported esophagogastrostomy and 1052 patients were involved [12, 14–19, 21, 23–25, 28, 31, 33]. The incidence of postoperative RE, stricture, anastomotic leakage, early complications and residual food were 27.3%, 12.5%, 2.2%, 15.2% and 41.4%, respectively. The incidence of RE was reported to be the highest among all reconstruction methods.

### Gastric tube reconstruction

Five studies reported outcomes of GT with 202 patients included [13, 14, 21, 32, 34]. The incidence of postoperative RE, stricture, anastomotic leakage, early complications, and residual food was 4.5%, 14.5%, 2.5%, 17.7% and 18.2%, respectively. The incidence of postoperative RE was the lowest, but anastomotic stricture was most frequently observed among all reconstruction methods.

### Jejunal interposition

A total of nineteen studies reported JI with 814 patients included [13, 16–18, 20–23, 25, 26, 28, 30–32, 34–38]. Incidences of postoperative outcomes such as RE, stricture, anastomotic leakage, early complications and residual food were 7.1%, 8.3%, 2.4%, 11.3% and 39.4%. The incidence of postoperative residual food was high.

### Jejunal pouch interposition

Three studies reported JPI with 43 patients included [18, 25, 30]. Incidence of postoperative RE, stricture, anastomotic leakage, early complications and residual food were 14.0%, 9.3%, 4.7%, 18.6% and 91.7%. The incidence of postoperative residual food was reported to be the highest among all methods, but only one study reported this index.

### Double flap technique

Only one comparative study investigated DFT and 14 patients were involved [20]. The incidence of postoperative RE, stricture, anastomotic leakage, early complications and residual food were 9.1%, 0%, 7.1%, 14.3% and 36.3%, respectively.

### Meta-analysis results

#### Esophagogastric anastomosis versus esophagojejunal anastomosis

**Fig. 2:**
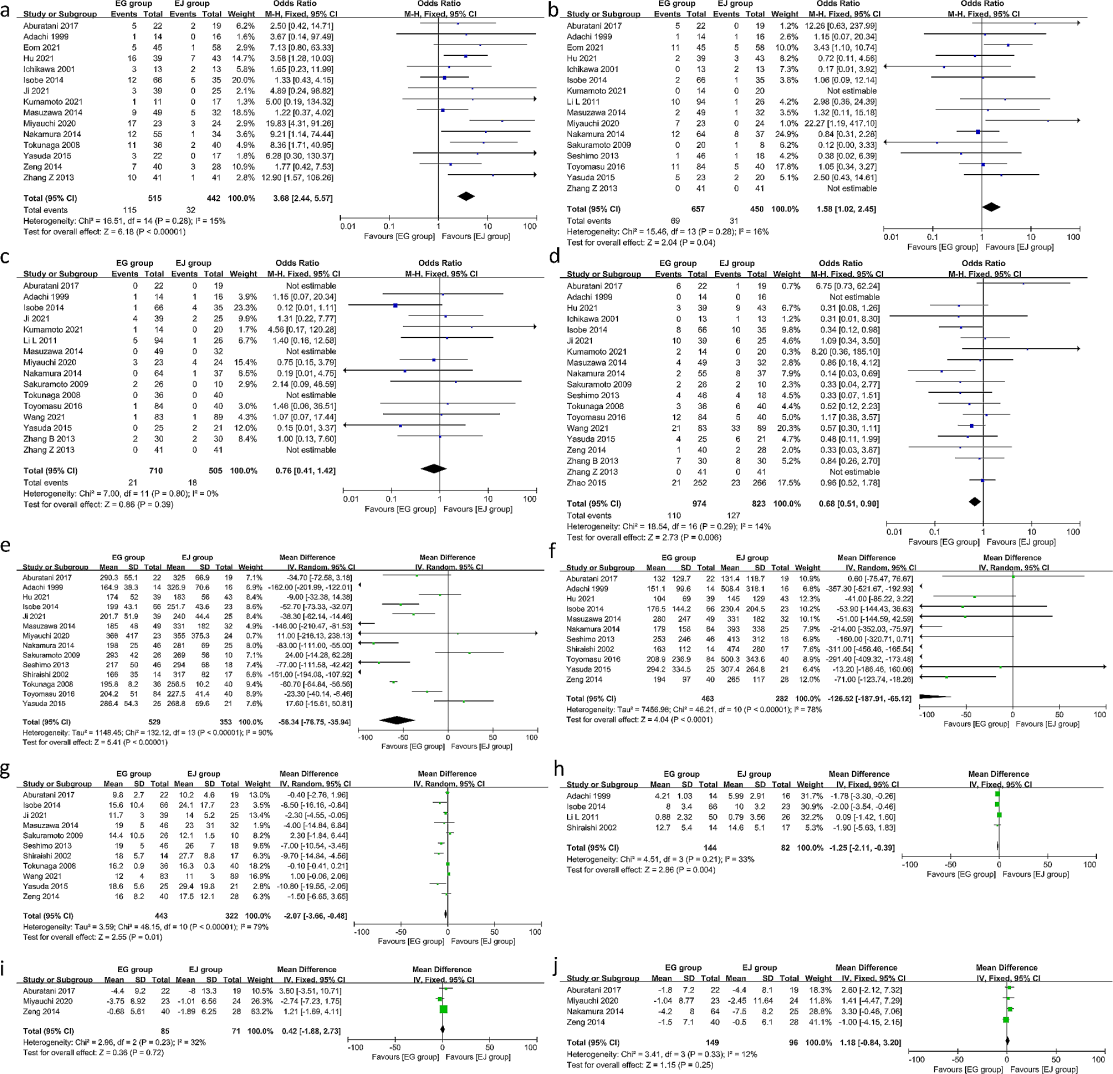
Forest plots between the EG group and EJ group. (**a**) reflux esophagitis (**b**) anastomotic stricture (**c**) anastomotic leakage (**d**) early complications (**e**) operation time (**f**) intraoperative blood loss (**g**) postoperative hospital (**h**) body weight loss (**i**) hemoglobin loss (**j**) albumin loss

Esophagogastric anastomosis group (EG group) included EG, GT and DFT, while esophagojejunal anastomosis group (EJ group) included DTR, JI and JPI. The pooled results showed that the incidence of RE was higher in the EG group than the EJ group (OR = 3.68, 95%CI 2.44–5.57, *P* < 0.00001, I^2^ = 15%). And the incidence of anastomotic stricture was higher in the EG group (OR = 1.58, 95%CI 1.02–2.45, *P* = 0.04, I^2^ = 16%). There was no significant difference in anastomotic leakage between the two groups (OR = 0.76, 95%CI 0.41–1.42, *P* = 0.39, I^2^ = 0%). Additionally, there were fewer early postoperative complications in the EG group (OR = 0.68, 95%CI 0.51–0.90, *P* = 0.006, I^2^ = 14%). Concerning the surgical indicators, the operation time was shorter (MD=-56.34, 95%CI -76.75- -35.94, *P* < 0.00001, I^2^ = 90%), the intraoperative blood loss was lesser (MD=-126.52, 95%CI -187.91- -65.12, *P* < 0.0001, I^2^ = 78%) and the postoperative hospital stay was shorter in the EG group (MD=-2.07, 95%CI -3.66- -0.48, *P* = 0.01, I^2^ = 79%). In terms of the nutritional indexes, the EG group experienced lesser weight loss 12 months after surgery than the EJ group (MD=-1.25, 95%CI -2.11- -0.39, *P* = 0.004, I^2^ = 33%). However, there was no significant difference between the two groups in hemoglobin (MD = 0.42, 95%CI -1.88- 2.73, *P* = 0.72, I^2^ = 32%) and albumin (MD = 1.18, 95%CI -0.84- 3.20, *P* = 0.25, I^2^ = 12%). The details are presented in Fig. 2.

#### DTR versus EG

**Fig. 3:**
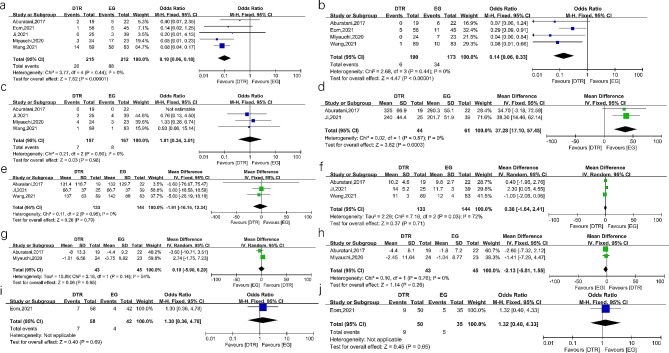
Forest plots between DTR and EG. (**a**) reflux esophagitis (**b**) anastomotic stricture (**c**) anastomotic leakage (**d**) operation time (**e**) intraoperative blood loss (**f**) postoperative hospital stays (**g**) changes in hemoglobin (**h**) changes in albumin (**i**) number of iron deficiency anemia (**j**) number of vitaminB12 deficiency

The pooled results showed that the incidences of RE (OR = 0.10, 95%CI 0.06–0.18, *P* < 0.00001, I^2^ = 0%) and anastomotic stricture (OR = 0.14, 95%CI 0.06–0.33, *P* < 0.00001, I^2^ = 0%) were lower in the DTR group. No significant difference was found in anastomotic leakage (OR = 1.01, 95%CI 0.34–3.01, *P* = 0.98, I^2^ = 0%). In terms of the surgical and postoperative recovery indicators, the operation time was shorter in EG (MD = 37.28, 95%CI 17.10-57.45, *P* = 0.0003, I^2^ = 0%). But the intraoperative blood loss and postoperative hospital stays (MD = 0.38, 95%CI -1.64-2.41, *P* = 0.71, I^2^ = 72%) were comparable between the two groups. In addition, there was no significant difference between two groups in hemoglobin, albumin, vitamin B12, and iron deficiency anemia. The details are presented in Fig. 3.

#### JI versus EG

**Fig. 4:**
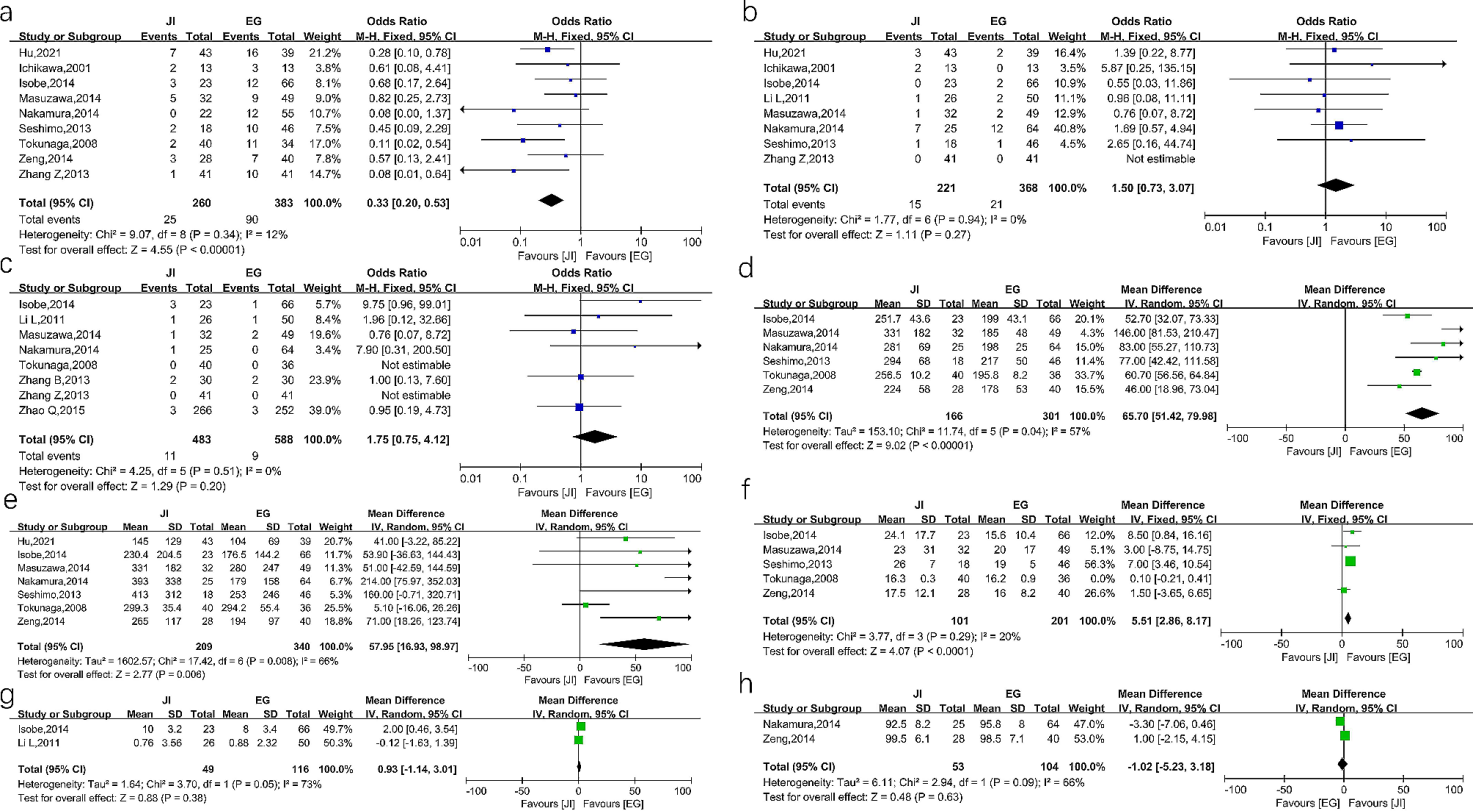
Forest plots between JI and EG. (**a**) reflux esophagitis (**b**) anastomotic stricture (**c**) anastomotic leakage (**d**) operation time (**e**) intraoperative blood loss (**f**) postoperative hospital stays (**g**) body weight loss (**h**) postoperative/ preoperative ratio of albumin

The results showed that there was fewer RE in the JI group (OR = 0.33, 95%CI 0.20–0.53, *P* < 0.00001, I^2^ = 12%). There was no significant difference between the two groups in anastomotic stricture (OR = 1.50, 95%CI 0.73–3.07, *P* = 0.27, I^2^ = 0%) and anastomotic leakage (OR = 1.75, 95%CI 0.75–4.12, *P* = 0.20, I^2^ = 0%). Concerning the surgical indicators, the operation time was significantly shorter in esophagogastrostomy (MD = 65.70, 95%CI 51.42–79.98, *P* < 0.00001, I^2^ = 57%). Furthermore, the intraoperative blood loss was lesser (MD = 57.95, 95%CI 16.93–98.97, *P* = 0.006, I^2^ = 66%) and the postoperative hospital stay was shorter in esophagogastrostomy (MD = 5.51, 95%CI 2.86–8.17, *P* < 0.0001, I^2^ = 20%). The weight loss and level of albumin 12 months after surgery were comparable in the two groups. These results are presented in Fig. 4. In terms of the long-term symptoms, the symptoms including diarrhea, dysphagia, heartburn, ileus and distention, did not differ significantly between the two groups. The reflux symptom was fewer in the JI group (OR = 0.29, 95%CI 0.14–0.59, *P* = 0.0006, I^2^ = 29%). The details of long-term symptoms are shown in Supplementary Table 1.

#### JI versus JPI

**Fig. 5:**
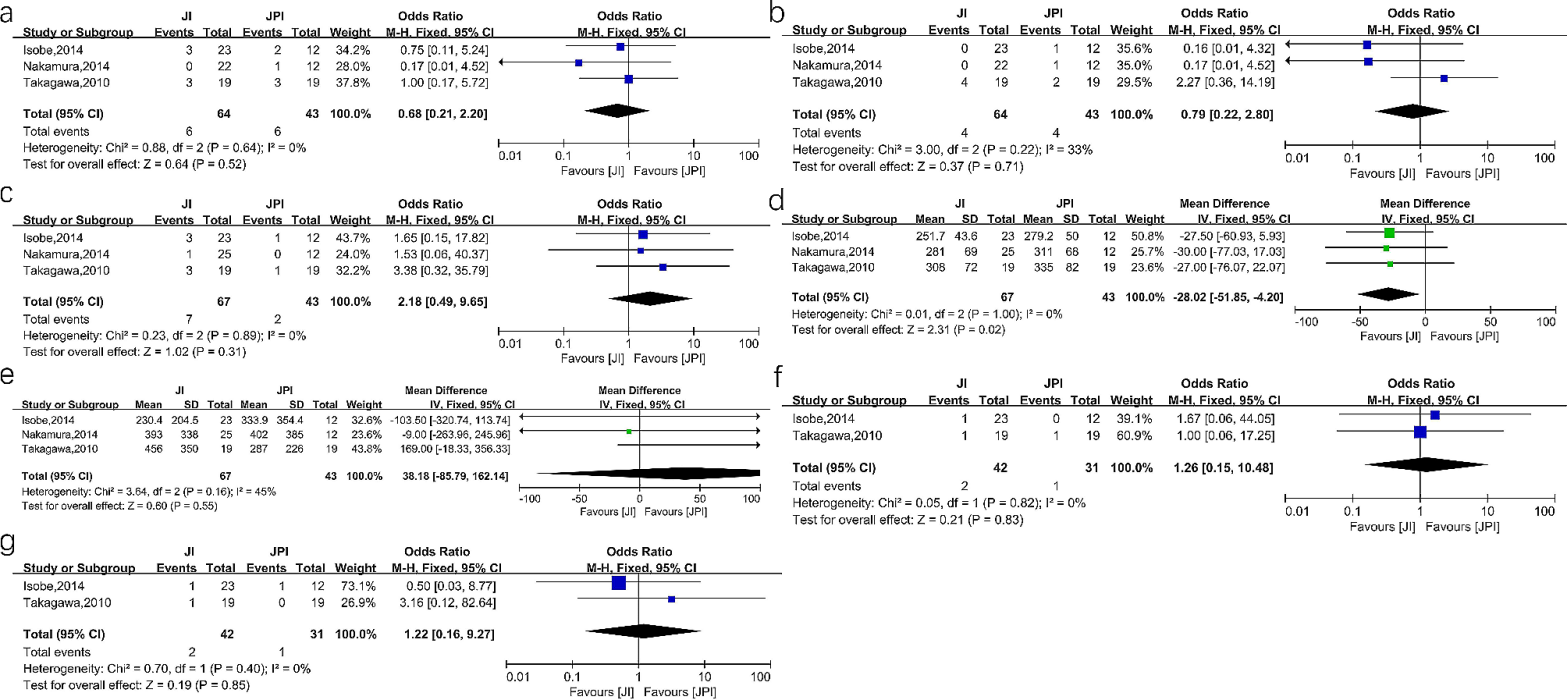
Forest plots between JI and JPI. (**a**) reflux esophagitis (**b**) anastomotic stricture (**c**) anastomotic leakage (**d**) operation time (**e**) intraoperative blood loss (**f**) long-term symptom of abdominal pain (**g**) long-term symptom of diarrhea

There was no significant difference in postoperative RE between the two groups (OR = 0.68, 95%CI 0.21–2.20, *P* = 0.52, I^2^ = 0%). In addition, no difference was found between the two groups regarding anastomotic stricture (OR = 0.79, 95%CI 0.22–2.80, *P* = 0.71, I^2^ = 33%) and anastomotic leakage (OR = 2.18, 95%CI 0.49–9.65, *P* = 0.31, I^2^ = 0%). Concerning the surgical indicators, the pooled results showed that operation time was shorter in the JI group (MD=-28.02, 95%CI -51.85- -4.20, *P* = 0.02, I^2^ = 41%). The intraoperative blood loss was similar in the two groups (MD = 38.18, 95%CI -85.79-162.14, *P* = 0.55, I^2^ = 45%). Additionally, two studies reported symptoms of abdominal pain and diarrhea 12 months after surgery [18, 30]. There was no significant difference in long-term symptoms between the two groups. The details of the results are showed in Fig. 5.

#### DTR versus JI

**Fig. 6:**
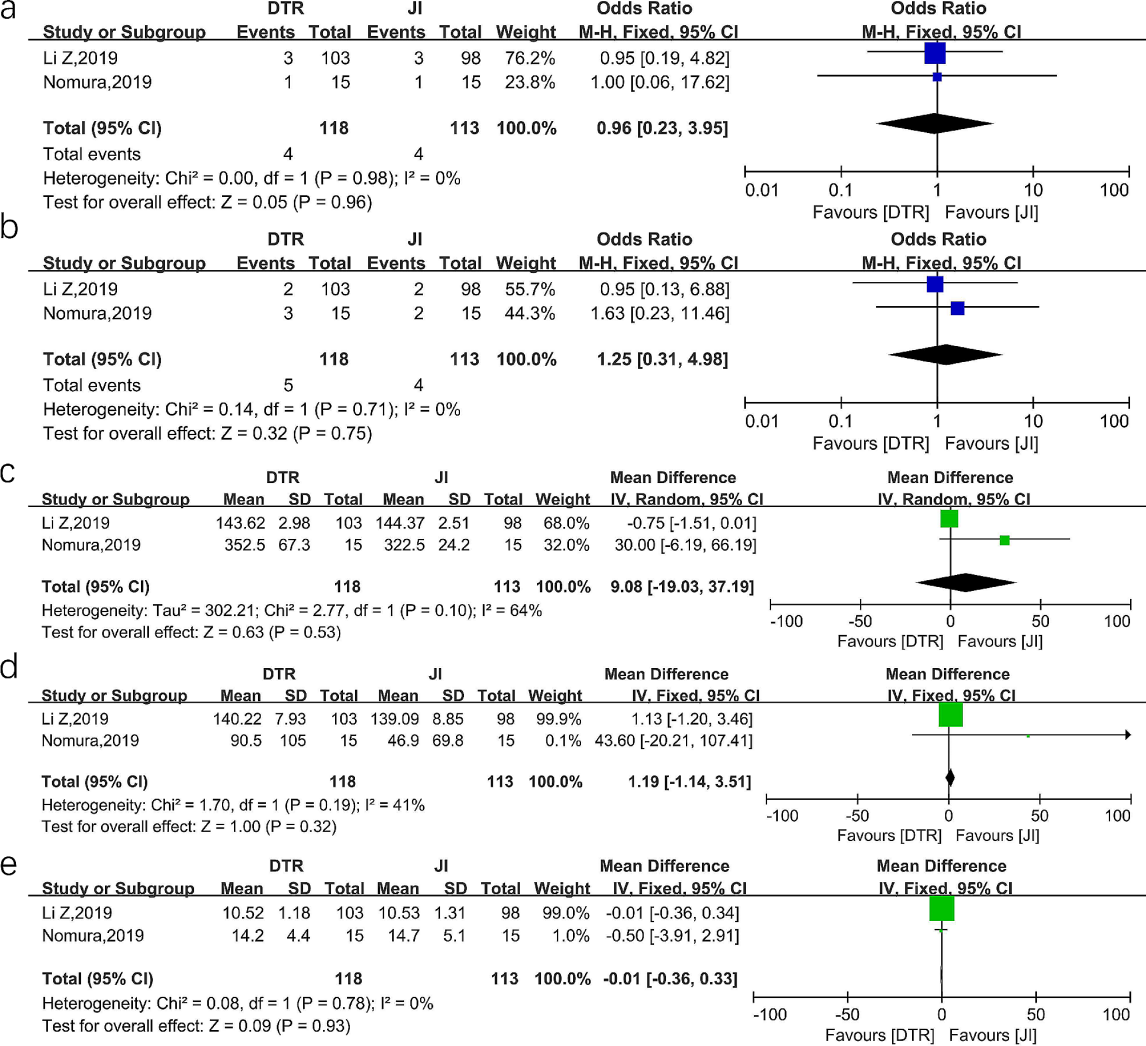
Forest plots between DTR and JI. (**a**) reflux esophagitis (**b**) early postoperative complications (**c**) operation time (**d**) intraoperative blood loss (**e**) postoperative hospital stays

Two studies reported outcomes comparing DTR and JI. There was no significant difference in RE (OR = 0.96, 95%CI 0.23–3.95, *P* = 0.96, I^2^ = 0%) and postoperative short-term complications (OR = 1.25, 95%CI 0.31–4.98, *P* = 0.75, I^2^ = 0%) in the two groups. In addition, there was no significant difference in operation time (MD = 9.08, 95%CI -19.03-37.19, *P* = 0.53, I^2^ = 64%), intraoperative blood loss (MD = 1.19, 95%CI -1.14-3.51, *P* = 0.32, I^2^ = 41%) and the postoperative hospital stay between the two groups (MD=-0.01, 95%CI -0.36-0.33, *P* = 0.93, I^2^ = 0%). These results are provided in Fig. 6.

#### JI versus GT

**Fig. 7:**
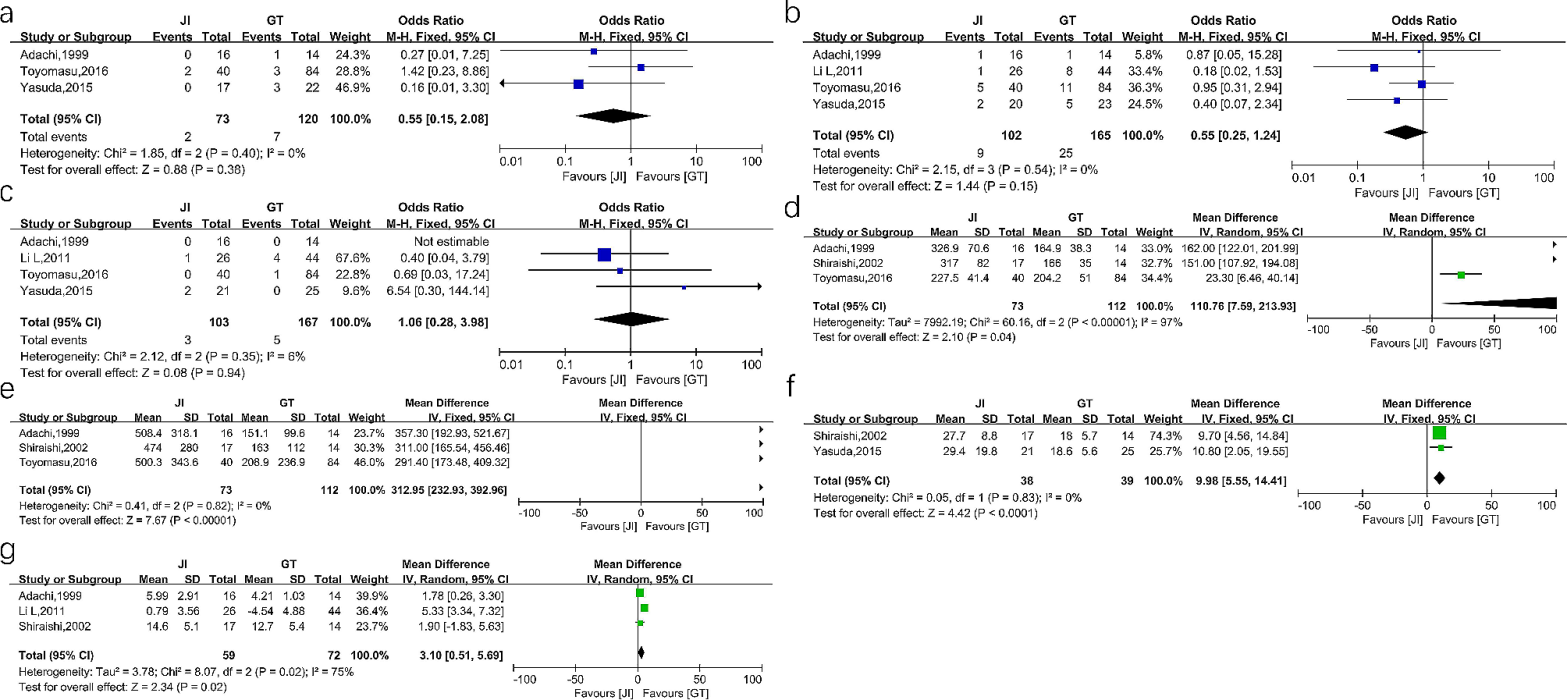
Forest plots between JI and GT. (**a**) reflux esophagitis (**b**) anastomotic stricture (**c**) anastomotic leakage (**d**) operation time (**e**) intraoperative blood loss (**f**) postoperative hospital stays (**g**) body weight loss

**Fig. 8:**
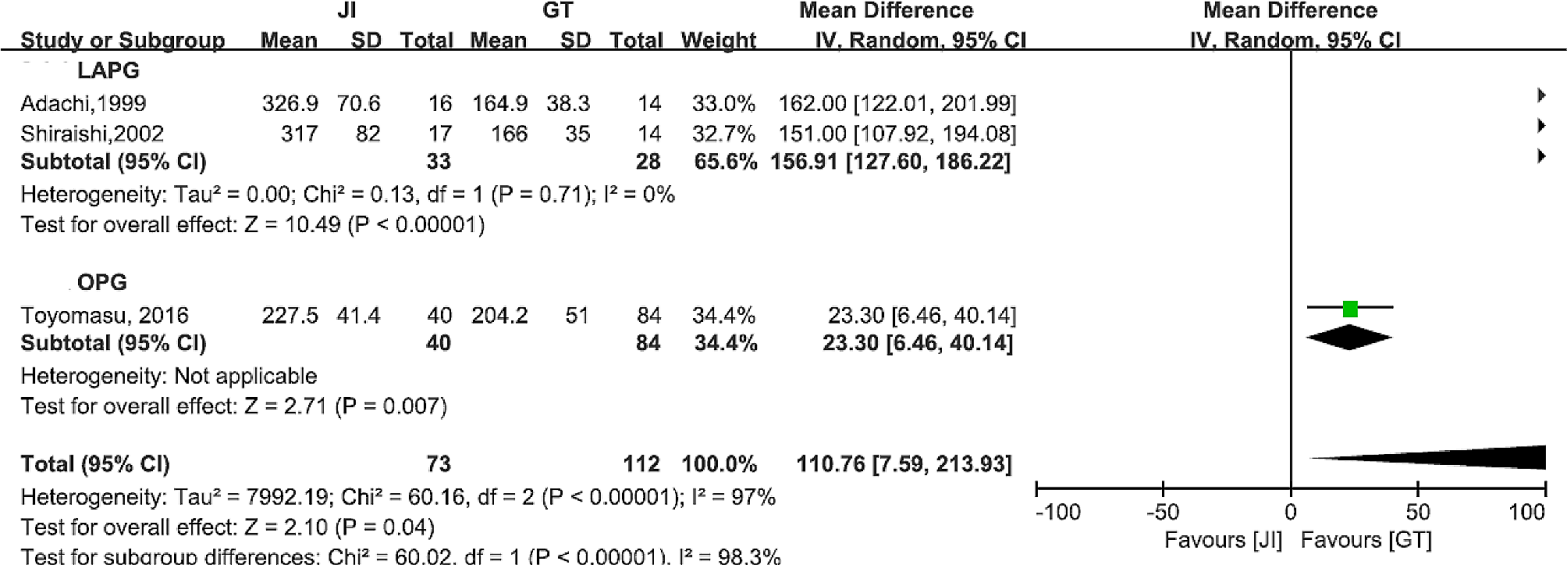
Subgroup analysis of operation time between JI and GT according to surgical approach

The incidence rates of postoperative RE (OR = 0.55, 95%CI 0.15–2.08, *P* = 0.38, I^2^ = 0%), anastomotic stricture (OR = 0.55, 95%CI 0.25–1.24, *P* = 0.15, I^2^ = 0%) and anastomotic leakage were comparable between the two groups (OR = 1.06, 95%CI 0.28–3.98, *P* = 0.94, I^2^ = 6%). The operation time of the GT group was significant shorter (MD = 110.76, 95%CI 7.59-213.93, *P* = 0.04, I^2^ = 97%). Additionally, the intraoperative blood loss was lesser (MD = 312.95, 95%CI 232.93-392.96, *P* < 0.00001, I^2^ = 0%) and the postoperative hospital stay was shorter in the GT group (MD = 9.98, 95%CI 5.55–14.41, *P* < 0.0001, I^2^ = 0%). In terms of body weight 12 months after surgery, we found that patients in the GT group experienced lesser weight loss than the JI group (MD = 3.10, 95%CI 0.51–5.69, *P* = 0.02, I^2^ = 75%). These results are shown in Fig. 7. To adjust the heterogeneity of operation time, we performed the subgroup analysis according to the surgical approach: LAPG and OPG. The results found no heterogeneity in LAPG subgroup (I^2^ = 0%, P _heterogeneity_=0.71). And only one study was included in the OPG subgroup. Subgroup analysis is shown in Fig. 8.

Quality and risk of bias assessment Supplementary Table 2 shows the quality of included 24 nonrandomized studies. All these studies scored well according to the NOS score. The results of risk of bias of RCTs are provided in Supplementary Figure 1. Publication bias was evaluated using a funnel plot, and the result is shown in Supplementary Figure 2.

## Discussion

In previous studies, PG could maintain comparable oncological radicalness to TG in EGC [39]. Moreover, in the KLASS 05 randomized clinical trial, patients who received PG showed better nutritional status and physical and social functions than patients received TG [40]. However, postoperative complications such as RE and anastomotic stricture may severely impair the long-term QoL after PG. Therefore, the effectiveness of anti-reflux, the incidence of postoperative complications and the QoL of various reconstruction methods after PG need to be compared urgently. By comprehensively searching the literature, a total of 27 comparative studies were identified and included in this systematic review, including 4 RCTs and 23 nonrandomized studies. The present study is an updated meta-analysis with a larger sample size to evaluate the short-term outcomes and long-term QoL of various reconstruction methods. To our best knowledge, this is the first systematic review and meta-analysis involving patients’ long-term QoL and nutritional status after PG. In addition, this is also the first meta-analysis that comprehensively included comparative studies concerning the reconstruction methods after PG.

This systematic review divided reconstruction methods into esophagogastric anastomosis (EG group) or esophagojejunal anastomosis (EJ group). We found that EJ group had advantages in decreasing the incidence of RE and stricture compared to the EG group. The previous studies showed that esophagogastric anastomotic sites were narrower and more likely to develop strictures than esophagojejunal anastomoses, which was consistent with our results [12]. However, due to the technique complexity, the EJ group required more operation time and presented more intraoperative blood loss and longer postoperative hospital stay than the EG group. Additionally, fewer early postoperative complications were observed in EG group. These results suggested that esophagogastric anastomosis and esophagojejunal anastomosis each had its advantages. Concerning the postoperative nutritional status, we found that the EG group had advantages in maintaining weight than the EJ group. The possible reason was that all the food passed through the stomach and duodenum passage in the EG group, and the nutrient substance could be absorbed fully. But there was no difference in hemoglobin and albumin 12 months after surgery between the two groups.

Esophagogastrostomy is a conventional method with technical simplicity advantages. The qualitative analysis showed that the incidence of anastomotic leakage of esophagogastrostomy was the lowest among all reconstruction methods, and the incidence of early postoperative complications was lower than DTR, JI and JPI. Moreover, the operation time was shorter and intraoperative blood loss was lesser. However, over one fourth of patients developed RE after esophagogastrostomy, which was highest in all reconstruction methods. And it showed a higher incidence rate of anastomotic stricture than DTR. Some previous studies showed that the incidence of RE could be reduced by modified operation, such as side overlap esophagogastrostomy and SPADE operation [41, 42]. However, the effectiveness of these modified operations remains to be confirmed by studies with larger sample size.

Many studies demonstrated that jejunal interposition was associated with a lower risk of RE. Our study showed that JI could significantly decrease the incidence of RE to 7.1% compared to EG. Despite the favorable anti-reflux effectiveness, the procedure was more technically complex, requiring three anastomoses. Compared to EG, the operation time was longer, the blood loss was more, and the postoperative hospital stay was also longer. However, no significant difference in postoperative leakage and early postoperative complications was found between JI and EG, which was consistent with the previous reports [43]. It might attribute to the skillful operation of surgeons and anastomosis performed by stapler under direct vision [21, 22]. In the present study, JPI had no notable advantages in RE, anastomotic stricture, and postoperative complications compared to JI. The operation time was longer in JPI. Additionally, by endoscopic examination, Nakamura et al. reported that residual food was revealed in 92% of patients after JPI, which might decrease the long-term QoL [25].

In terms of DTR, our results showed an excellent effectiveness in anti-reflux, with the incidence of RE decreasing to 7.6%. Compared to esophagogastrostomy, DTR showed lower rates of anastomotic stricture. In terms of surgical safety, although DTR needed three anastomoses and longer operation time, there was no significant difference in postoperative complications between esophagogastrostomy and DTR. These results suggested that DTR was a safe technique after PG. Compared to JI, DTR adds a side-to-side anastomosis between remnant stomach and jejunum, effectively avoiding emptying dysfunction. In addition, we found that the residual food was least in DTR among all reconstruction methods. Regarding postoperative nutrition, we found no difference in hemoglobin, albumin, vitamin B12, and iron between esophagogastrostomy and DTR. Two studies previously reported the long-term QoL of DTR [15, 19]. Ji reported that DTR had apparent advantages over esophagogastrostomy based on EORTC QLQ-C30 and EORTC QLQ-STO22 questionnaires [19]. To sum up, DTR is a safe technique with excellent anti-reflux effectiveness and long-term QoL.

In the present study, GT showed a good effectiveness in reducing RE. In qualitative analysis, only 4.5% of patients presented RE which was the lowest in all methods. However, the anastomotic stricture occurred in 14.5% patients who underwent GT, which was highest. Moreover, only a few studies with small sample sizes investigated this reconstruction method, and the effectiveness remains to be confirmed.

In the previous study, Shoji et al. reported a 4.2% of incidence rate of RE after the DFT [44]. A meta-analysis also demonstrated that DFT could lower the rates of complications compared to DTR, EG, JI and JPI [43]. However, all four studies included in the meta-analysis were single-arm studies. By comprehensive searching, only one comparative study investigating DFT was included in the present study.

There were several limitations in the present study. First, most of the studies included were retrospective and nonrandomized studies. This certainly attenuated the evidence level. Second, in terms of postoperative nutritional status, some original data could not be obtained. And the questionnaires for evaluating long-term QoL were quite inconsistent between studies, so some long-term indexes could not be compared. Third, all the studies included in the present study were reported by Asian authors. The results need further confirmation in other countries. Fourth, the reconstruction techniques might be related to the incidence of postoperative complications. However, no studies included in the meta-analysis provided the comparative data in terms of the reconstruction techniques.

## Conclusion

Esophagojejunal anastomosis after proximal gastrectomy can reduce the reflux esophagitis and anastomotic stricture incidences, while esophagogastric anastomosis has advantages in technical simplicity and long-term weight status. Various reconstruction methods have advantages over esophagogastrostomy in reducing postoperative reflux esophagitis. Jejunal interposition and jejunal pouch interposition increase the technical complexity and the risk of postoperative residue food, while gastric tube reconstruction shows a high incidence of anastomotic stricture. Double tract reconstruction is a safe technique with excellent anti-reflux effectiveness and favorable long-term QoL. The effectiveness of other reconstruction methods, such as the double flap technique, need more prospective studies to confirm.

### Electronic supplementary material

Below is the link to the electronic supplementary material.

Supplementary Material 1

Supplementary Material 2

Supplementary Material 3

Supplementary Material 4

## Data Availability

The datasets used and/or analyzed during the current study are available from the corresponding author on reasonable request.

## Abbreviations

PRISMA: Reporting Items for Systematic Reviews and Meta-analyses
DTR: Double tract reconstruction
EG: Esophagogastrostomy
GT: Gastric tube reconstruction
JI: Jejunal interposition
JPI: Jejunal pouch interposition
DFT: Double flap technique
RE: Reflux esophagitis
GC: Gastric cancer
EGC: Early gastric cancer
PG: Proximal gastrectomy
QoL: Quality of life
AEG: Adenocarcinoma of esophagogastric junction
TG: Total gastrectomy
MeSH: Medical Subject Headings
NOS: Newcastle-Ottawa quality assessment scale
RCT: Randomized controlled trail
SOFY: Side overlap with fundoplication by Yamashita
SPADE: Spade-shaped anastomosis
