# Supplementary material for: Short-term outcomes and long-term quality of life of reconstruction methods after proximal gastrectomy: a systematic review and meta-analysis
Source: BMC Cancer. 2024 Jan 10;24:56. doi: 10.1186/s12885-024-11827-4 (PMC10777503; doi:10.1186/s12885-024-11827-4)
Supplement: Supplementary file 1 — Supplementary Material 1 [file 12885_2024_11827_MOESM1_ESM.docx]

**Supplementary Table 1.** The postoperative long-term symptoms between JI and EG.

|  |  | | Sample size | |  |  |  |  |
| --- | --- | --- | --- | --- | --- | --- | --- | --- |
| Complaints | | No.of studies | JI | EG | Heterogeneity  (I^2^, P) | OR | 95% CI | P |
| Diarrhea | | 4 | 126 | 194 | 0%, 0.71 | 2.46 | 0.72-8.40 | 0.15 |
| Dysphagia | | 2 | 66 | 105 | 0%, 0.51 | 1.90 | 0.64-5.57 | 0.25 |
| Reflux | | 3 | 94 | 145 | 29%, 0.24 | 0.29 | 0.14-0.59 | 0.0006 |
| Heartburn | | 3 | 86 | 139 | 0%, 0.87 | 0.77 | 0.35-1.67 | 0.50 |
| Ileus | | 2 | 60 | 89 | NA, NA | 1.44 | 0.09-24.11 | 0.80 |
| Distention | | 2 | 66 | 105 | 0%, 0.58 | 2.42 | 1.05-5.56 | 0.04 |
