# Supplementary material for: Short-term outcomes and long-term quality of life of reconstruction methods after proximal gastrectomy: a systematic review and meta-analysis
Source: BMC Cancer. 2024 Jan 10;24:56. doi: 10.1186/s12885-024-11827-4 (PMC10777503; doi:10.1186/s12885-024-11827-4)
Supplement: Supplementary file 2 — Supplementary Material 2 [file 12885_2024_11827_MOESM2_ESM.docx]

**Supplementary Table 2.** Newcastle-Ottawa quality assessment scale.

| Author | Is the case definition adequate? | Representativeness of the cases | Selection of controls | Definition of controls | Comparability of cases and controls on the basis of the design and analysis | Ascertainment of exposure | Same method of ascertainment for cases and controls | Non-response rate | Total score |
| --- | --- | --- | --- | --- | --- | --- | --- | --- | --- |
| Zhang B, 2013 | ★ | ☆ | ★ | ★ | ★☆ | ★ | ★ | ☆ | 6 |
| Zhao Q, 2015 | ★ | ☆ | ★ | ★ | ★★ | ★ | ★ | ☆ | 7 |
| Wang X, 2021 | ★ | ☆ | ★ | ★ | ★★ | ★ | ★ | ★ | 8 |
| Eom,2021 | ★ | ☆ | ★ | ★ | ★☆ | ★ | ★ | ☆ | 6 |
| Ji X, 2021 | ★ | ☆ | ★ | ★ | ★★ | ★ | ★ | ☆ | 7 |
| Nomura E,2019 | ★ | ☆ | ★ | ★ | ★☆ | ★ | ★ | ★ | 7 |
| Nakamura ,2014 | ★ | ☆ | ★ | ★ | ★☆ | ★ | ★ | ★ | 7 |
| Chen X,2012 | ★ | ★ | ★ | ★ | ★★ | ★ | ★ | ☆ | 8 |
| Zeng C,2014 | ★ | ★ | ★ | ★ | ★★ | ★ | ★ | ☆ | 8 |
| Li L,2011 | ★ | ☆ | ★ | ★ | ★☆ | ★ | ★ | ★ | 7 |
| Yasuda A,2015 | ★ | ☆ | ★ | ★ | ★★ | ★ | ★ | ☆ | 7 |
| Tokunaga,2008 | ★ | ☆ | ★ | ★ | ★★ | ★ | ★ | ☆ | 7 |
| Adachi Y,1999 | ★ | ☆ | ★ | ★ | ★☆ | ★ | ★ | ★ | 7 |
| Miyaochi,2020 | ★ | ☆ | ★ | ★ | ★☆ | ★ | ★ | ☆ | 6 |
| Masuzawa T,2014 | ★ | ★ | ★ | ★ | ★★ | ★ | ★ | ☆ | 8 |
| Isobe T,2014 | ★ | ☆ | ★ | ★ | ★☆ | ★ | ★ | ☆ | 6 |
| Aburatani T,2017 | ★ | ★ | ★ | ★ | ★★ | ★ | ★ | ★ | 9 |
| Toyomasu,2017 | ★ | ★ | ★ | ★ | ★★ | ★ | ★ | ☆ | 8 |
| Kumamoto ,2021 | ★ | ★ | ★ | ★ | ★☆ | ★ | ★ | ☆ | 7 |
| Hu L,2021 | ★ | ☆ | ★ | ★ | ★☆ | ★ | ★ | ☆ | 6 |
| Seshimo,2013 | ★ | ★ | ★ | ★ | ★☆ | ★ | ★ | ☆ | 7 |
| Ichikawa D, 2001 | ★ | ★ | ★ | ★ | ★☆ | ★ | ★ | ☆ | 7 |
| Sakuramoto,2009 | ★ | ★ | ★ | ★ | ★☆ | ★ | ★ | ☆ | 7 |
| Eom B, 2021 | ★ | ☆ | ★ | ★ | ★☆ | ★ | ★ | ☆ | 6 |
