# Supplementary figures and images for: Short-term outcomes and long-term quality of life of reconstruction methods after proximal gastrectomy: a systematic review and meta-analysis
Source: BMC Cancer. 2024 Jan 10;24:56. doi: 10.1186/s12885-024-11827-4 (PMC10777503; doi:10.1186/s12885-024-11827-4)

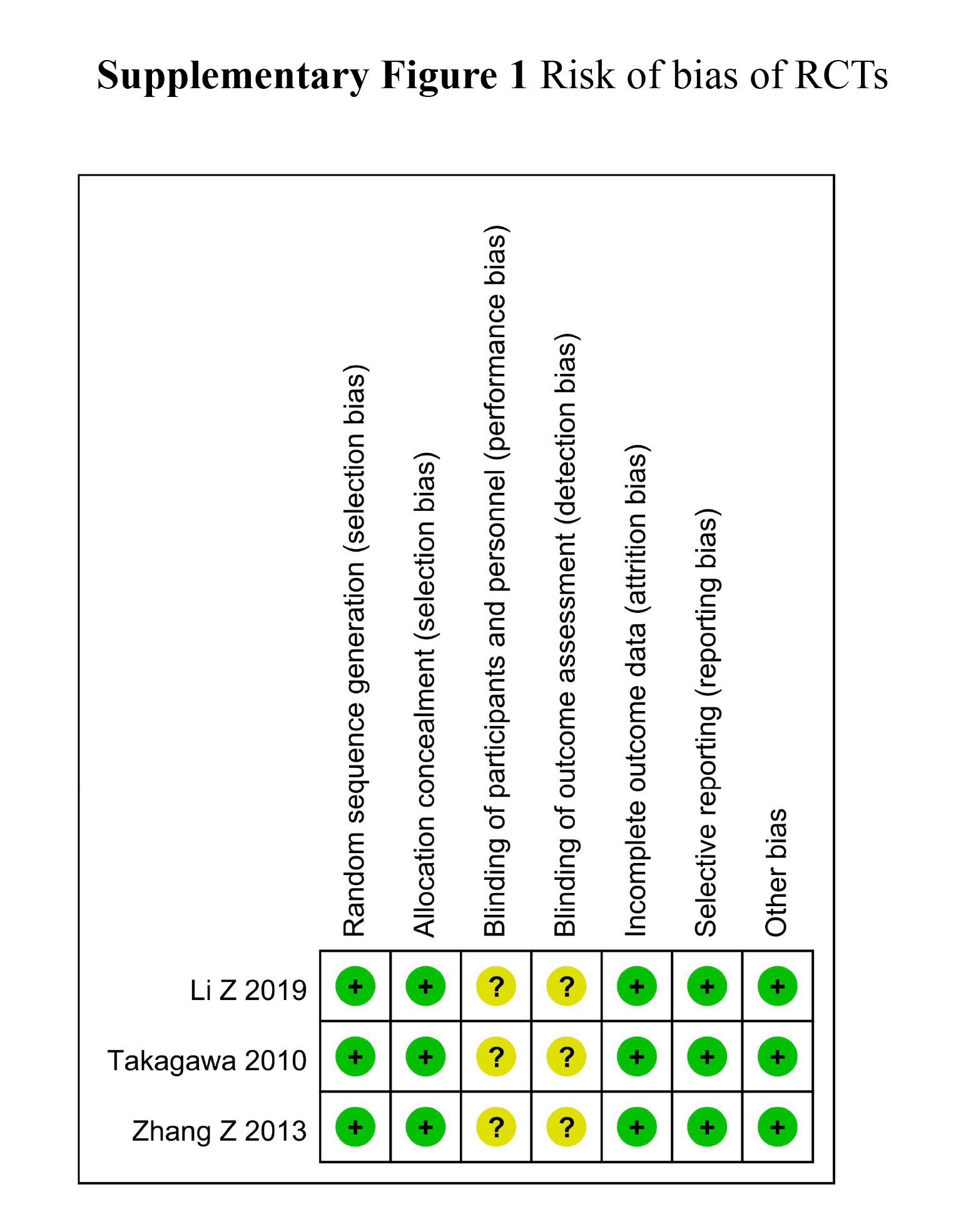

Supplement: Supplementary file 3 — Supplementary Material 3 [file 12885_2024_11827_MOESM3_ESM.tif]

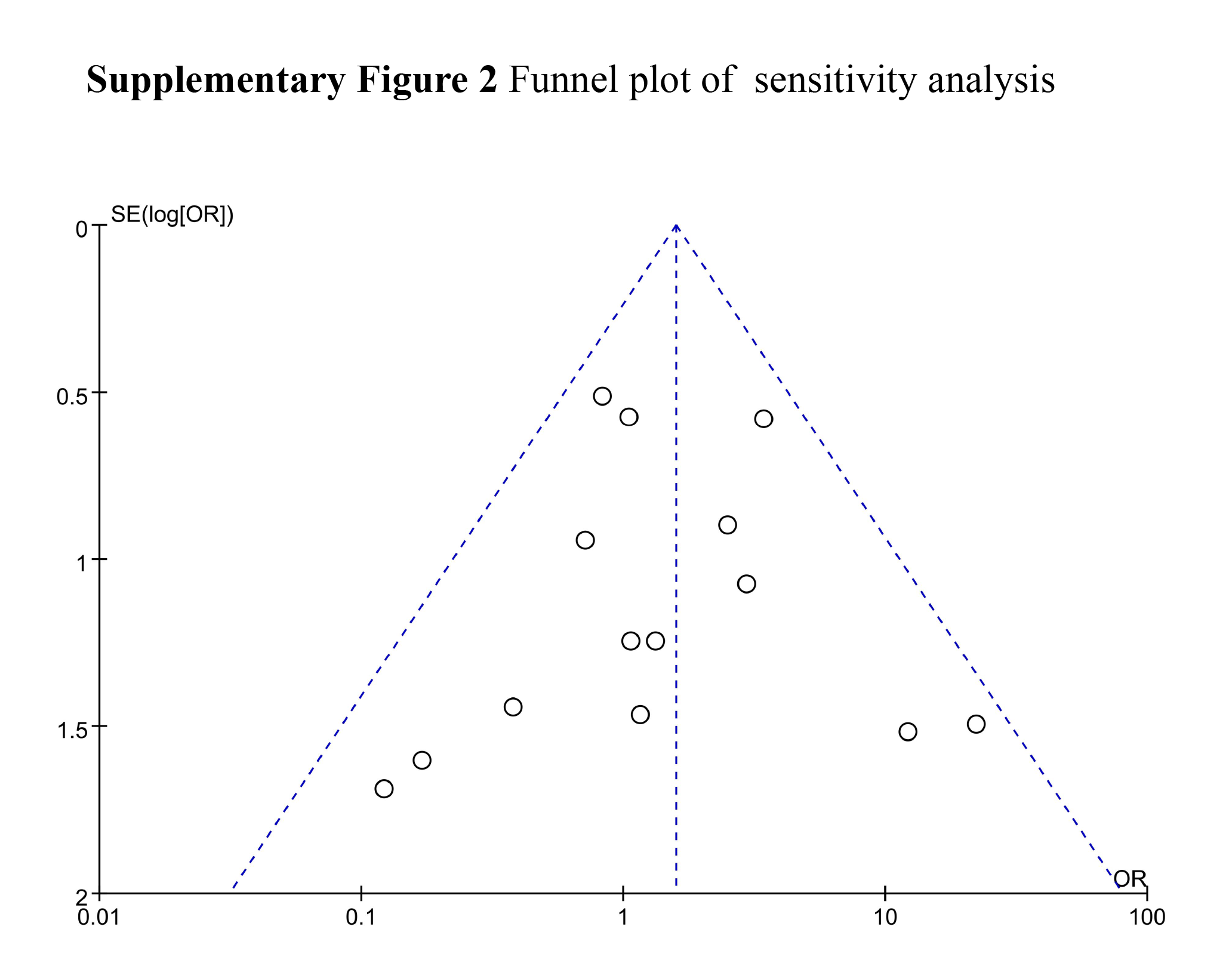

Supplement: Supplementary file 4 — Supplementary Material 4 [file 12885_2024_11827_MOESM4_ESM.tif]
